# Phantom-based evaluation of radiomics feature stability for low-dose CT lung cancer screening

**DOI:** 10.3389/fendo.2026.1880461

**Published:** 2026-07-21

**Authors:** Sunyi Zheng, Xiaomeng Yang, Hongren Wang, Zhipeng Gao, Pengchun Ye, Weiping Wang, Wenhua Li, Donghua Meng, Shuai Zhang, Wenjia Zhang, Houpu Liu, Shuyuan Huang, Chunlin Zhang, Jing Wang, Jihui Hao, Xiaonan Cui

**Affiliations:** 1 Department of Radiology, Medical Artificial General Intelligence for Computation (MAGIC) Lab, National Clinical Research Center for Cancer, Tianjin’s Clinical Research Center for Cancer, State Key Laboratory of Druggability Evaluation and Systematic Translational Medicine, Tianjin Key Laboratory of Digestive Cancer, Tianjin Key Laboratory of Cancer Prevention and Therapy, Tianjin Medical University Cancer Institute and Hospital, Tianjin, China; 2 Department of Radiology, Huadu District People’s Hospital of Guangzhou, Guangzhou, Guangdong, China; 3 Academy of Medical Engineering and Translational Medicine, Tianjin University, Tianjin, China; 4 National Clinical Research Center for Cancer, Tianjin’s Clinical Research Center for Cancer, State Key Laboratory of Druggability Evaluation and Systematic Translational Medicine, Tianjin Key Laboratory of Digestive Cancer, Tianjin Key Laboratory of Cancer Prevention and Therapy, Tianjin Medical University Cancer Institute and Hospital, Tianjin, China; 5 Department of Radiology, Ordos Central Hospital, Ordos, China; 6 Department of Public Health, Erasmus Medical Center Rotterdam, Rotterdam, Netherlands; 7 Department of Radiology, The Second Hospital of Shanxi Medical University, Taiyuan, China; 8 School of Public Health, Tianjin University of Traditional Chinese Medicine, Tianjin, China; 9 Pancreas Center, Tianjin Medical University Cancer Institute and Hospital, National Clinical Research Center for Cancer, State Key Laboratory of Druggability Evaluation and Systematic Translational Medicine, Tianjin Key Laboratory of Digestive Cancer, Tianjin’s Clinical Research Center for Cancer, Tianjin, China

**Keywords:** computed tomography, feature stability, lung cancer screening, phantom, radiomics

## Abstract

**Objective:**

To assess the stability of radiomic features derived from lung nodules under low-dose CT lung cancer screening conditions and to evaluate the influence of feature stability on model performance.

**Methods:**

A chest phantom containing eight simulated lung nodules was scanned on five CT scanners using varying tube voltages (100–120 kVp) and tube currents (20–60 mA·s) to simulate inter-scanner and intra-scanner variability. Nodule radiomic features were extracted accordingly and their stability was evaluated using the intraclass correlation coefficient. Stable features were grouped by hierarchical clustering, from which representative stable features were selected for each cluster. Models constructed using representative stable features and remaining unstable features were compared in two independent lung cancer screening datasets for nodule malignancy assessment and growth prediction. Model performance was evaluated using the area under the receiver operating characteristic curve (AUC).

**Results:**

Inter-scanner variability had a greater impact on feature stability than intra-scanner variability, with tube current exerting more influence than tube voltage. Models based on representative stable features achieved better performance than those using unstable features in both the malignancy assessment cohort (AUC, 0.995 vs 0.945) and nodule growth prediction cohort (AUC, 0.799 vs 0.610). Models using representative stable features also showed smaller performance differences between training and test sets than those using unstable features.

**Conclusion:**

Radiomic features with high stability under lung cancer screening conditions were associated with improved performance consistency in independent screening datasets. These findings suggest stability-informed feature selection may contribute to more reliable radiomics applications in lung cancer screening.

## 1 Introduction

Lung cancer remains the leading cause of cancer-related mortality worldwide, with approximately 2.48 million new cases and 1.82 million deaths reported in 2022 (1, 2). This burden has driven extensive investigation across multiple fields, including the molecular mechanisms underlying tumor progression (3–5), novel therapeutic strategies and drug delivery systems targeting non-small cell lung cancer (6, 7), and pharmacoeconomic evaluations of emerging first-line regimens (8). Given this context, low-dose computed tomography (LDCT) has been increasingly used in lung cancer screening. Landmark randomized trials have demonstrated its mortality benefit in high-risk populations. The National Lung Screening Trial reported a 20% reduction in lung cancer mortality with LDCT compared with chest radiography (9). A finding further confirmed by the Dutch-Belgian Randomized Lung Cancer Screening Trial, which showed a 24% mortality reduction among male participants (10). Despite these advances, challenges in pulmonary nodule characterization and risk stratification persist, highlighting the need for more refined quantitative imaging approaches to optimize screening performance and clinical decision-making.

In lung cancer screening, radiomics serves as an important approach for pulmonary nodule analysis by transforming imaging data into mineable, high-dimensional features, thereby enabling quantitative and objective assessment. Prior studies have shown that radiomic features derived from screening CT can characterize tumor growth patterns and biological behavior. For example, Lu et al. showed that multi-window CT radiomic features could be used to distinguish indolent from aggressive lung cancers in a screening setting, outperforming single-window approaches (11). More recently, leveraging longitudinal imaging information, Wang et al. developed a serial low-dose CT-based radiomics reinforcement learning model to improve early lung cancer diagnosis and risk stratification in screening populations (12).

While radiomics has shown encouraging results in lung cancer screening, the stability of radiomic features remains a key challenge for clinical translation. In particular, variability in feature selection has been reported across studies addressing the same screening objective, such as malignant nodule prediction. For this application, Liu et al. identified predictive features dominated by texture descriptors derived from Laplacian-of-Gaussian filtering and wavelet transformation (13). By contrast, Zyla et al. reported a different set of selected features, including GLCM Inverse Variance, Shape Flatness, and GLCM Id, which are non-filtered radiomics features reflecting fundamental texture and shape properties (14). Nevertheless, partial agreement in feature selection has also been reported. Despite differences in patient cohorts, Selvam et al. and Shi et al. found overlapping radiomic signatures, with morphological features such as sphericity retained (15, 16). Previous studies have investigated the impact of scanner manufacturers, imaging platforms, and CT acquisition parameters on radiomic feature stability in routine clinical and diagnostic settings (17–21). However, in lung cancer screening settings, radiomic feature stability and the clinical relevance of modeling stable radiomic features have not been explored. Furthermore, chest LDCT screening frequently encounters incidental endocrine lesions within the scanned volume—most notably adrenal incidentalomas, detected in approximately 2% of screening participants (22). While CT-based radiomic characterization is increasingly investigated to differentiate benign from malignant adrenal masses (23, 24), such endocrine applications face similar acquisition-dependent reproducibility challenges due to cross-scanner variability.

Therefore, this study aimed to assess the stability of radiomic features derived from lung nodules under low-dose CT lung cancer screening conditions by systematically accounting for variability arising from inter-scanner protocol differences and intra-scanner parameter fluctuations. The study was also designed to examine the influence of radiomic feature stability on model performance by comparing models constructed using stable versus unstable features in two independent lung cancer screening datasets.

## 2 Materials and methods

### 2.1 Study design

**Figure 1:**
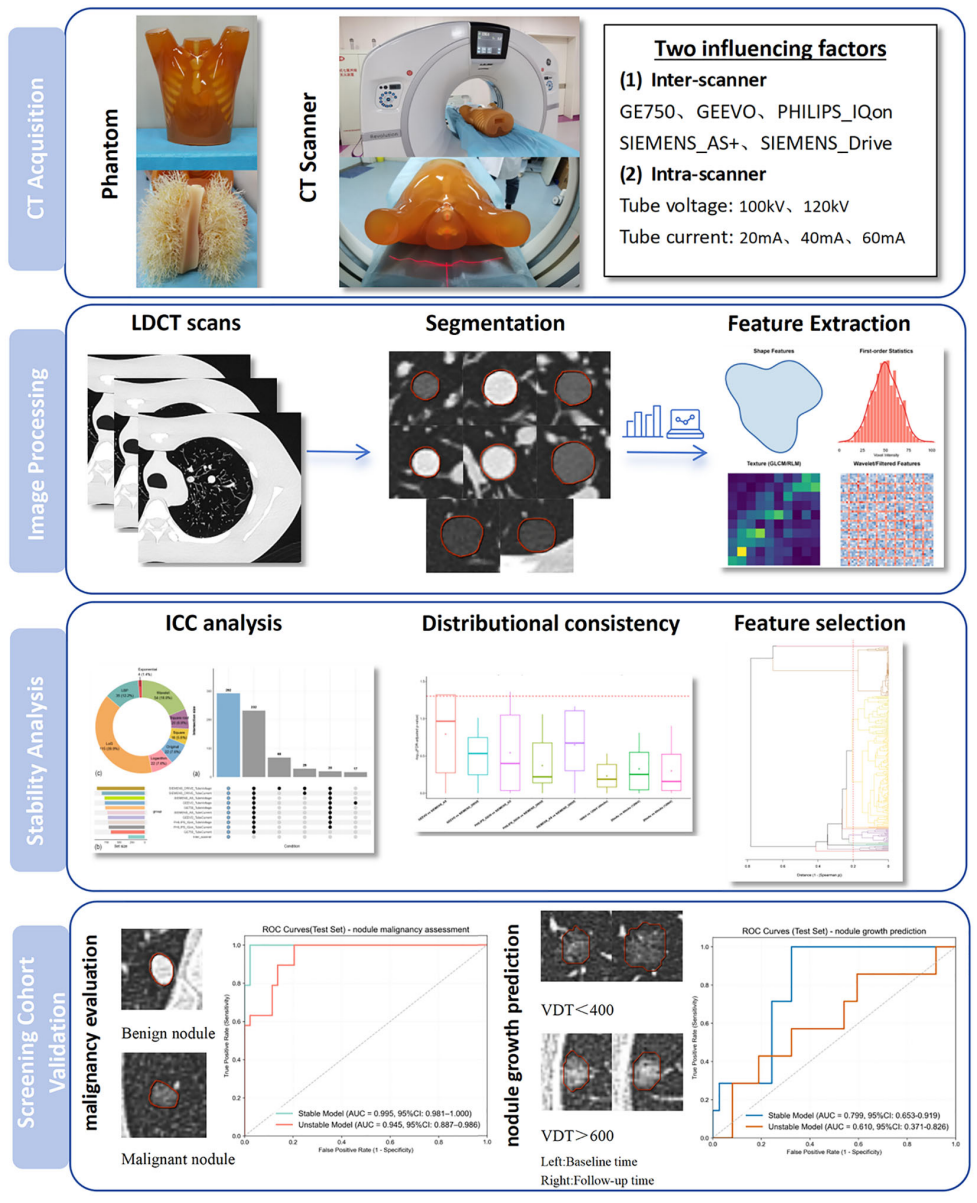
Overview of the study workflow. The study workflow consisted of four main stages: CT acquisition, image processing, stability analysis, and validation. A phantom containing eight nodules with different densities was scanned on five CT scanners using lung cancer screening acquisition parameters, after which radiomics features were extracted. Feature stability was assessed using intraclass correlation coefficients, distribution consistency analysis, and clustering. The selected stable features were subsequently used for malignancy assessment and nodule growth prediction, and their clinical utility was evaluated through comparison with models constructed using the remaining unstable features. VDT, volume doubling time; GE750, Discovery CT750 HD (GE); GEEVO, Revolution EVO (GE); PHILIPS_IQon, IQon Spectral CT (Philips); SIEMENS_AS+, SOMATOM Definition AS+ (Siemens); SIEMENS_Drive, SOMATOM Drive (Siemens).

Institutional review board approval was obtained for this retrospective study, and the requirement for written informed consent was waived. This study included a phantom-based experiment to investigate the stability of radiomics features, followed by validation in two retrospective lung cancer screening datasets. The overall study workflow is illustrated in **Figure 1**. Specifically, a phantom containing lung nodules with different densities was scanned on CTs using lung cancer screening acquisition parameters, after which radiomics features of nodules were extracted. Feature stability was evaluated in inter-scanner and intra-scanner tests. Inter-scanner variability reflects differences among CT scanner manufacturers, whereas intra-scanner variability is attributable to variations in CT acquisition parameters including tube voltage and tube current. After radiomics feature stability analysis, representative stable features and the remaining unstable features were identified. Models based on these features were constructed separately for malignancy assessment and nodule growth prediction to evaluate their clinical utility.

### 2.2 Lung phantom

The study used the LUNGMAN N1 multipurpose anthropomorphic chest phantom (43×40×48 cm; 18 kg; chest circumference, 94 cm). This life-size model was manufactured by Kyoto Kagaku (Kyoto, Japan; purchased in 2021). It replicates the anatomy of a healthy adult male thorax, with soft-tissue and lung X-ray attenuation closely matching human values. Internal components such as the pulmonary vessels, trachea, heart, mediastinum, and selected abdominal structures can be removed individually.

The phantom contained eight simulated solid and ground-glass lung nodules (25–27). These comprised solid nodules with a density of 100 HU and diameters of 8, 10, and 12 mm; ground-glass nodules with a density of −630 HU in the same diameter range; and ground-glass nodules with a density of −800 HU and diameters of 10 and 12 mm. The eight nodules were distributed across the upper, middle, and lower lung regions of the phantom.

### 2.3 CT acquisition

In the inter-scanner test, five CT scanners from multiple vendors were used. The CT scanners included Discovery CT750 HD and Revolution EVO from GE Healthcare, IQon Spectral CT from Philips Healthcare, and SOMATOM Definition AS+ and SOMATOM Drive from Siemens Healthineers. Owing to vendor-dependent differences in acquisition and reconstruction settings, the use of identical CT protocols across scanners was not feasible. Therefore, each scanner followed its routine lung cancer screening protocol with fixed CT acquisition parameters for analysis, which served as the scanner-specific baseline protocol. The corresponding acquisition parameters used in the inter-scanner test are summarized in **Table 1**.

**Table 1:** CT acquisition protocols in the inter-scanner test.

CT scanner (manufacturer)	Pitch*	Rotation times (s)	Tube voltage (kVp)	Tube current (mA·s)	FOV (mm)	Slice thickness (mm)*	Slice interval (mm)*	Reconstruction Kernel†	Reconstruction method†
Discovery CT750 HD (GE)	0.984375	0.5	120	40	400	1.25	1.25	LUNG	PLUS
Revolution EVO(GE)	0.984375	0.5	120	40	400	1.25	1.25	LUNG	PLUS
IQon Spectral CT (Philips)	1.015	0.5	120	40	400	1.5	1.5	YB	Lung
SOMATOM Definition AS+ (Siemens)	1	0.5	120	40	400	1.5	1.5	I70f	Lung
SOMATOM Drive (Siemens)	1	0.5	120	40	400	1.5	1.5	I70f	Lung

The number of decimal places was aligned with the display precision of the corresponding CT scanner control console.

* Pitch, slice thickness, and slice interval were limited to discrete values for each CT scanner; the closest available value was used as the baseline.

† Because reconstruction kernels vary across manufacturers, the kernel routinely used in lung cancer screening CT protocols for each scanner was selected as the baseline. The recommended reconstruction method for thoracic imaging was adopted for each scanner to maintain optimal image quality.

In the intra-scanner test, tube voltage and tube current were varied individually, while all other acquisition parameters were kept constant at the scanner-specific baseline settings. All five scanners were included to evaluate the magnitude of parameter-induced variability within each system. In accordance with lung cancer screening guideline recommendations for low-dose CT (28, 29), six combinations of tube voltage at two levels (100 and 120 kVp), and tube current at three levels (20, 40, and 60 mA·s) were applied for each scanner. The corresponding acquisition parameter combinations used in the intra-scanner test are summarized in **Table 2**.

**Table 2:** Acquisition parameter combinations in the intra-scanner test.

Tube voltage (kVp)	Tube current (mA·s)
100	20
100	40
100	60
120	20
120	40
120	60

### 2.4 Image segmentation and feature extraction

Semi-automatic segmentation of eight simulated lung nodules and extraction of radiomic features were performed using the Deepwise Multimodal Scientific Research Platform (Version 2.5.2, https://keyan.deepwise.com; Beijing Deepwise and League of PHD Technology Co., Ltd, Beijing, China) (30–33). A radiologist with five years of clinical experience performed region-of-interest segmentation for all nodules. The same radiologist repeated the segmentation after a three-week interval. Segmentation consistency was assessed using the Dice similarity coefficient, and a Dice score greater than 0.95 was achieved for all images across the two segmentations. Accordingly, radiomic features were extracted from the initial segmentation for subsequent feature selection analyses. The extracted features comprised first-order features, shape features, gray level co-occurrence matrix (GLCM) features, gray level size zone matrix (GLSZM) features, gray level run length matrix (GLRLM) features, gray level dependence matrix (GLDM) features, and neighborhood gray tone difference matrix (NGTDM) features. In total, 2022 radiomic features were obtained through this procedure.

### 2.5 Stability analysis

The intraclass correlation coefficient (ICC) with a two-way mixed-effects model for absolute agreement in single measurements [ICC (1, 2)] was used to evaluate radiomic feature reproducibility under inter-scanner and intra-scanner parameter variations. Features with ICC values of at least 0.95 and corresponding *p* values below 0.05 across all inter-scanner and intra-scanner assessments were defined as stable features, whereas features not meeting these criteria were designated as unstable features. To reduce feature redundancy, hierarchical clustering based on Spearman correlation was performed on the stable features, using a distance metric defined as 1 minus the absolute correlation coefficient and a clustering threshold of 0.2. Within each cluster, one representative feature was selected using the maximum median absolute deviation criterion to generate the final representative stable feature subset.

### 2.6 Validation in lung cancer screening datasets

After identifying the representative stable features and the unstable features, we evaluated their clinical relevance and predictive value for malignancy assessment and nodule growth pattern prediction in two independent screening datasets. In each cohort, patients were randomly divided into training and testing sets at a 7:3 ratio. For unstable feature sets, feature selection was performed on the training set using Spearman correlation analysis with a threshold of |*r*| ≥ 0.85 to remove highly correlated features, followed by cross-validated least absolute shrinkage and selection operator regression for optimal feature selection. The selected features were then used to construct predictive models based on the support vector machine (SVM) algorithm. In contrast, representative stable features, which had been identified through prior stability analysis and were limited in number, were directly used to build SVM-based models without additional feature selection. All SVM models used an RBF kernel (C = 0.5, class weights balanced for label imbalance), with features standardized on the training set before fitting. The optimal decision threshold was selected by the Youden Index, and AUC 95% CIs were estimated by 1,000-iteration bootstrap resampling. Furthermore, each representative stable feature was individually evaluated for its predictive performance, and the associations with the binary outcome were assessed using the Mann-Whitney U test in each of the two independent validation datasets.

Two retrospectively collected datasets from the lung cancer screening study at Tianjin Medical University Cancer Institute and Hospital were used for validation (34). The first dataset for nodule malignancy assessment comprised 207 patients with lung nodules (116 men and 91 women; mean age, 66.1 ± 7.0 years), including 143 benign and 64 malignant cases, collected between September 2018 and October 2019. The second dataset for nodule growth prediction was retrospectively collected and comprised 145 patients with pulmonary nodules (83 men and 62 women; mean age, 65.4 ± 7.3 years) from September 2018 to June 2019. This dataset comprised 25 nodules with a volume doubling time (VDT) < 400 days and 120 nodules with a VDT > 600 days. VDT was calculated from volumetric changes in the same nodule between two low-dose CT examinations in the same patient, with the initial scan serving as the baseline (35, 36). The patient inclusion and exclusion criteria are detailed in **Supplementary Material 1**. Detailed distributions of characteristics for the training and test sets in the two screening datasets are presented in **Supplementary Table 1**.

### 2.7 Statistical analysis

Statistical analyses were conducted using R software (version 4.5.2) and Python (version 3.9.23). Paired two-sided Wilcoxon signed-rank tests with a significance level of 0.05 were applied to characterize feature distributional variability by assessing pairwise differences between inter-scanner device pairs and intra-scanner acquisition parameter condition pairs. Model performance was assessed using receiver operating characteristic (ROC) analysis, quantified by the area under the curve (AUC). Statistical significance was defined as *p* < 0.05, with multiple comparisons adjusted using the false discovery rate (FDR) method.

## 3 Results

### 3.1 Feature stability in inter- and intra-scanner tests

**Figure 2:**
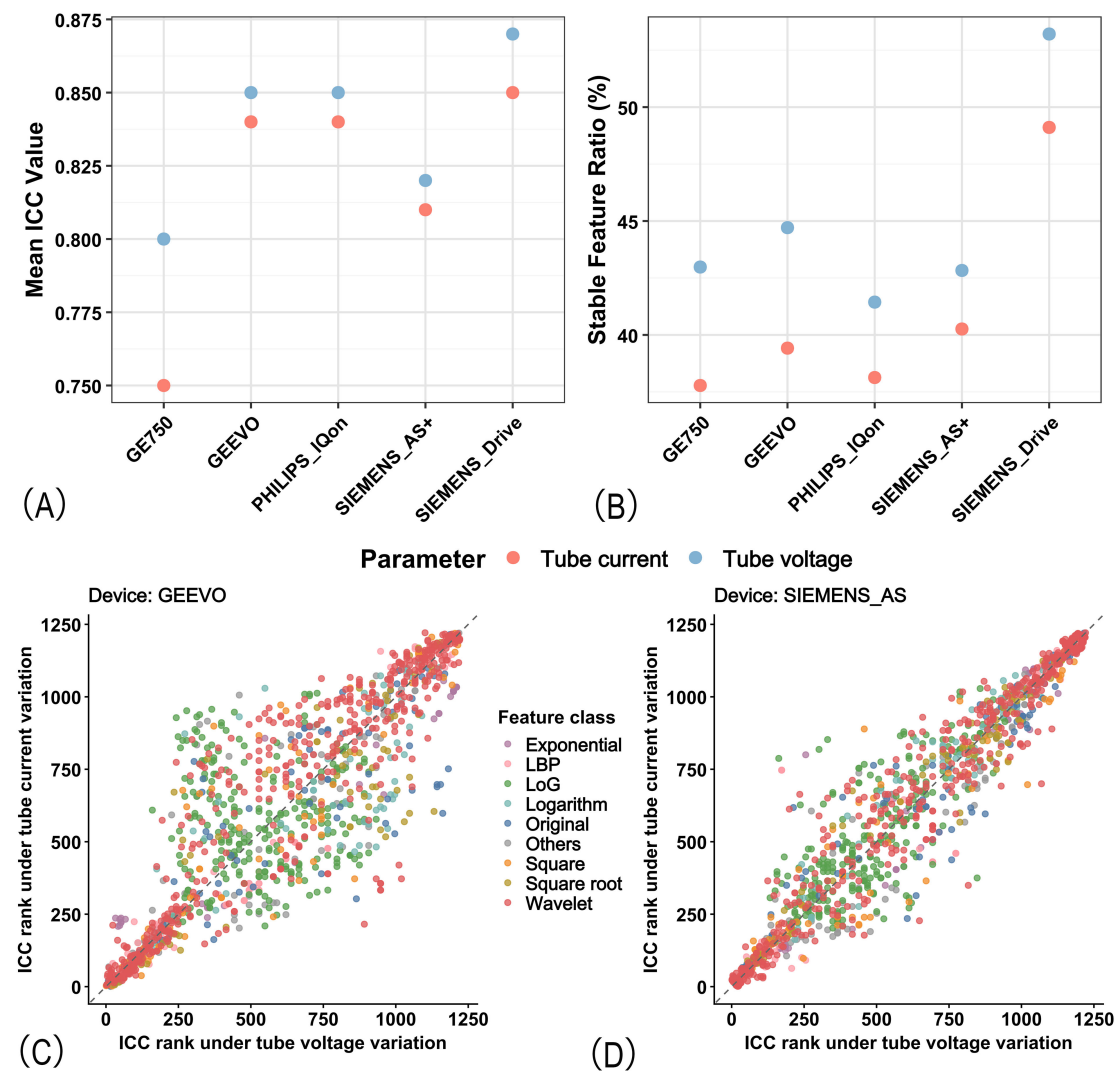
**(A)** Mean ICC of stable features under intra-scanner tube voltage and tube current variation for each scanner. **(B)** Proportion of stable features within the total feature set under intra-scanner parameter variation. **(C)** Feature-level ICC rank comparison in the Revolution EVO (GE) scanner. **(D)** Feature-level ICC rank comparison in the SOMATOM Definition AS+ (Siemens) scanner. Each point reflects one radiomic feature, with its position defined by the ICC-based robustness ranks associated with the two parameter variations. ICC, intraclass correlation coefficient; Stable features are defined as those with ICC ≥ 0.95, *p* < 0.05; GE750, Discovery CT750 HD (GE); GEEVO, Revolution EVO (GE); PHILIPS_IQon, IQon Spectral CT (Philips); SIEMENS_AS+, SOMATOM Definition AS+ (Siemens); SIEMENS_Drive, SOMATOM Drive (Siemens).

Radiomic feature stability across and within scanners is summarized in **Table 3**. The results indicate that inter-scanner stability (mean ICC, 0.67 ± 0.28; stable feature ratio, 16.07%) was lower than intra-scanner stability, with all intra-scanner conditions yielding mean ICCs of at least 0.75 and stable feature ratios of 37.78% or higher. Among intra-scanner acquisition parameters, radiomic feature stability was consistently greater for variations in tube voltage than for tube current, as evidenced by higher mean ICCs and a larger proportion of stable features (**Figures 2A, B**). In the Revolution EVO (GE) and SOMATOM Definition AS+ (Siemens) scanners, tube voltage and tube current presented a consistent impact pattern, with ICC-based rankings closely aligned along the 45-degree identity line (**Figures 2C, D**). The results indicated that radiomic features exhibiting high ICC values under tube voltage variation also tended to show high ICC values under tube current variation, and vice versa. Discovery CT750 HD (GE) and SOMATOM Drive (Siemens) also showed consistent impact patterns and details are provided in **Supplementary Figure 1**.

**Table 3:** Radiomics feature stability in inter- and intra-scanner tests.

Test		ICC Value*	Ratio†
Inter-scanner		0.67± 0.28	325/2022 (16.07%)
Intra-scanner			
Discovery CT750 HD (GE)	Tube voltage	0.80± 0.27	869/2022 (42.98%)
	Tube current	0.75± 0.30	764/2022 (37.78%)
Revolution EVO (GE)	Tube voltage	0.85± 0.20	904/2022 (44.71%)
	Tube current	0.84± 0.20	797/2022 (39.42%)
IQon Spectral CT (Philips)	Tube voltage	0.85± 0.21	838/2022 (41.44%)
	Tube current	0.84± 0.19	771/2022 (38.13%)
SOMATOM Definition AS+ (Siemens)	Tube voltage	0.82± 0.26	866/2022 (42.83%)
	Tube current	0.81± 0.25	814/2022 (40.26%)
SOMATOM Drive (Siemens)	Tube voltage	0.87± 0.21	1076/2022 (53.21%)
	Tube current	0.85± 0.23	993/2022 (49.11%)

*Values are presented as mean ± standard deviation for features.

†Ratios represent the proportion of stable features (ICC ≥ 0.95, *p* < 0.05) retained following segmentation consistency analysis.

ICC, intraclass correlation coefficient.

### 3.2 Distributional consistency of stable radiomics features with high ICC values

**Figure 3:**
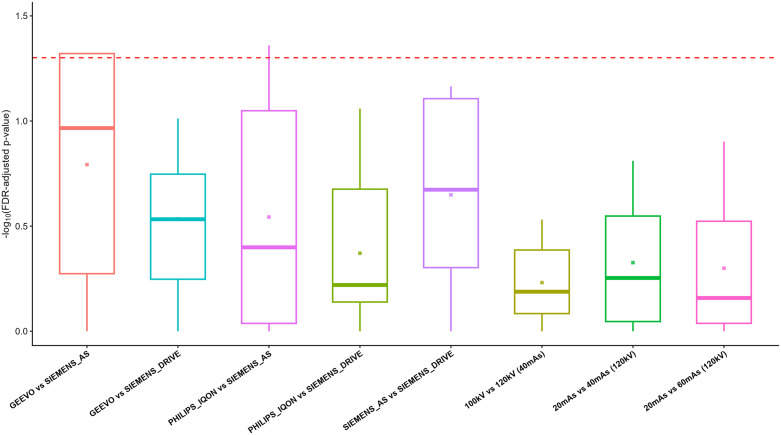
Distributional stability of radiomics features high ICC values (ICC ≥ 0.95) across representative inter- and intra-scanner comparisons. Boxplots illustrate the distribution of –log_10_(FDR–adjusted *p*–values) derived from paired two-sided Wilcoxon signed-rank tests for radiomics features with high ICC values. Five representative inter-scanner comparisons and three intra-scanner acquisition parameter comparisons are shown in the figure. The horizontal dashed line indicates the FDR-corrected significance threshold (α= 0.05). GEEVO, Revolution EVO (GE); PHILIPS_IQON, IQon Spectral CT (Philips); SIEMENS_AS, SOMATOM Definition AS+ (Siemens); SIEMENS_Drive, SOMATOM Drive (Siemens).

To further assess the distributional consistency of radiomic features with high ICC values (ICC ≥ 0.95), feature distributions were compared across inter-scanner pairs and intra-scanner acquisition parameter variations. **Figure 3** presents five representative inter-scanner pairwise comparisons together with paired intra-scanner analyses involving tube voltage and tube current variations. For inter-scanner evaluations, the vast majority of features (95%) showed no significant distributional differences across scanner pairs after multiple-comparison correction, with only 5% of feature-scanner comparisons exhibiting marginal statistical significance. Among these, features derived from the Laplacian of Gaussian (LoG) category accounted for the largest proportion (42.68%), followed by wavelet-based features (23.17%), with adjusted *p* values close to the significance threshold. In intra-scanner analyses performed on the Discovery CT750 HD scanner, no radiomic feature had a significant distributional difference across variations in tube voltage or tube current after correction. Similar nonsignificant findings were observed in the other five inter-scanner comparisons shown in **Supplementary Figure 2**.

### 3.3 Representative stable features

**Figure 4:**
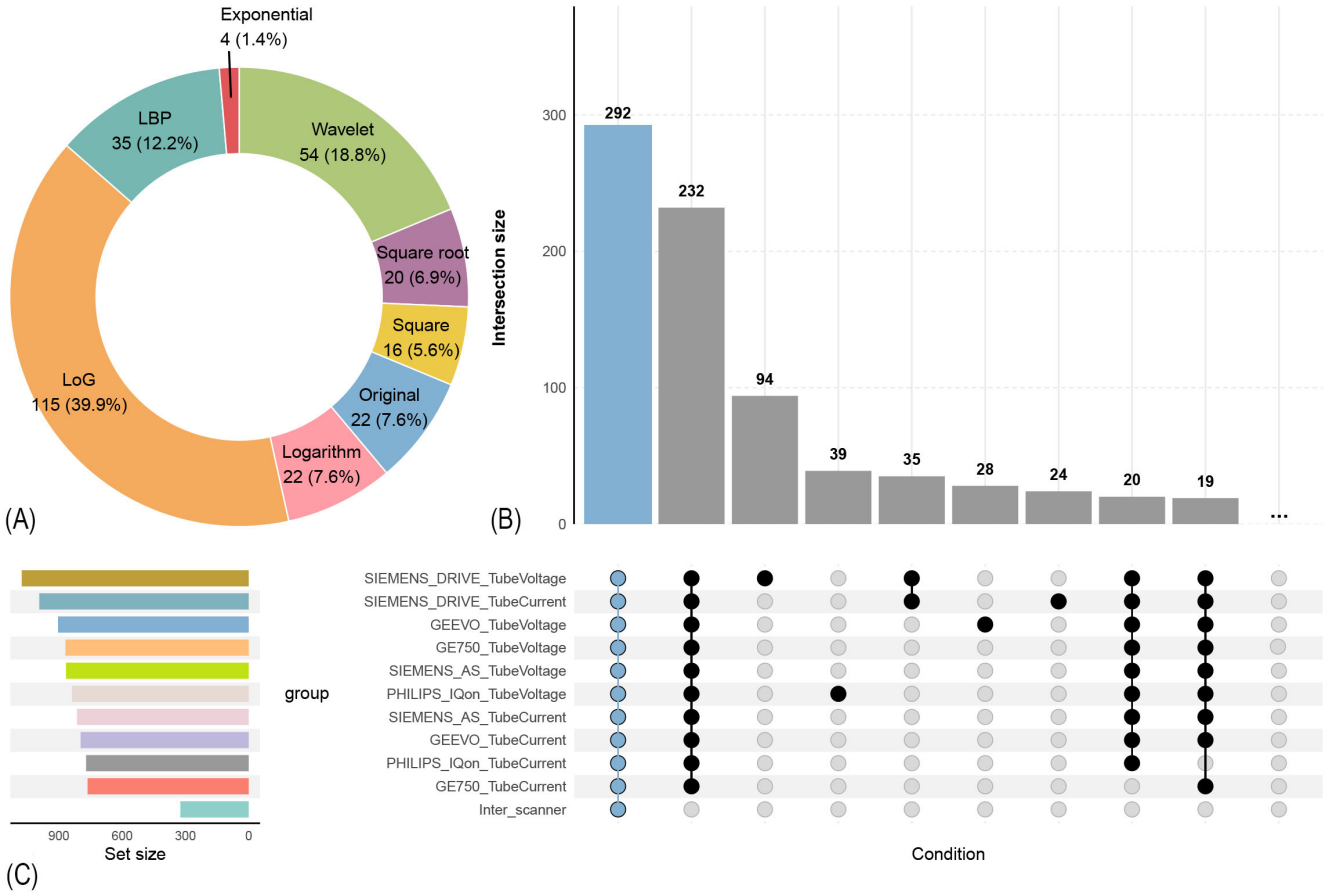
Distribution of stable radiomic features. **(A)** Donut chart. The chart illustrates the compositional distribution of the 292 stable features across different transformation categories, with embedded values indicating the number of features (and corresponding percentages) in each category. **(B)** Venn diagram. Each bar in the diagram denotes the number of radiomic features that were stable in the group corresponding to the highlighted section at the bottom, but unstable in the other groups. **(C)** Bar chart. This chart quantifies the total count of stable radiomic features in each test group. GE750, Discovery CT750 HD (GE); GEEVO, Revolution EVO (GE); PHILIPS_IQon, IQon Spectral CT (Philips); SIEMENS_AS, SOMATOM Definition AS+ (Siemens); SIEMENS_Drive, SOMATOM Drive (Siemens); LoG, Laplacian of Gaussian; LBP, local binary pattern.

The intersection of inter-scanner and intra-scanner analyses identified 292 stable radiomic features shown in **Figure 4**. These features were categorized by transformation type: original (22/292, 7.6%), logarithm (22/292, 7.6%), LoG (115/292, 39.9%), local binary pattern (LBP, 35/292, 12.2%), exponential (4/292, 1.4%), wavelet (54/292, 18.8%), square root (20/292, 6.9%), and square (16/292, 5.6%) (**Figure 4A**). Using hierarchical clustering (**Supplementary Figure 3**) and Max-Median Absolute Deviation-based selection, 13 representative radiomic features were identified from the 292 stable features, spanning original features as well as wavelet-, logarithm-, and log-sigma-transformed feature categories. The detailed feature names are provided in **Table 4**.

**Table 4:** Detailed information on 13 representative stable radiomic features.

Filter	Representative stable radiomic features
Original	original_firstorder_TotalEnergy
Wavelet	wavelet-LLH_firstorder_TotalEnergy
Logarithm	logarithm_glrlm_RunLengthNonUniformity
	logarithm_glcm_Idmn
	logarithm_glrlm_GrayLevelNonUniformity
	logarithm_firstorder_Mean
Log-sigma	log-sigma-4-0-mm-3D_glszm_GrayLevelNonUniformity
	log-sigma-4-0-mm-3D_ngtdm_Contrast
	log-sigma-3-0-mm-3D_firstorder_10Percentile
	log-sigma-3-0-mm-3D_glcm_ClusterProminence
	log-sigma-5-0-mm-3D_ngtdm_Complexity
	log-sigma-2-0-mm-3D_glcm_ClusterShade
	log-sigma-2-0-mm-3D_glcm_Imc1

**Figure 5:**
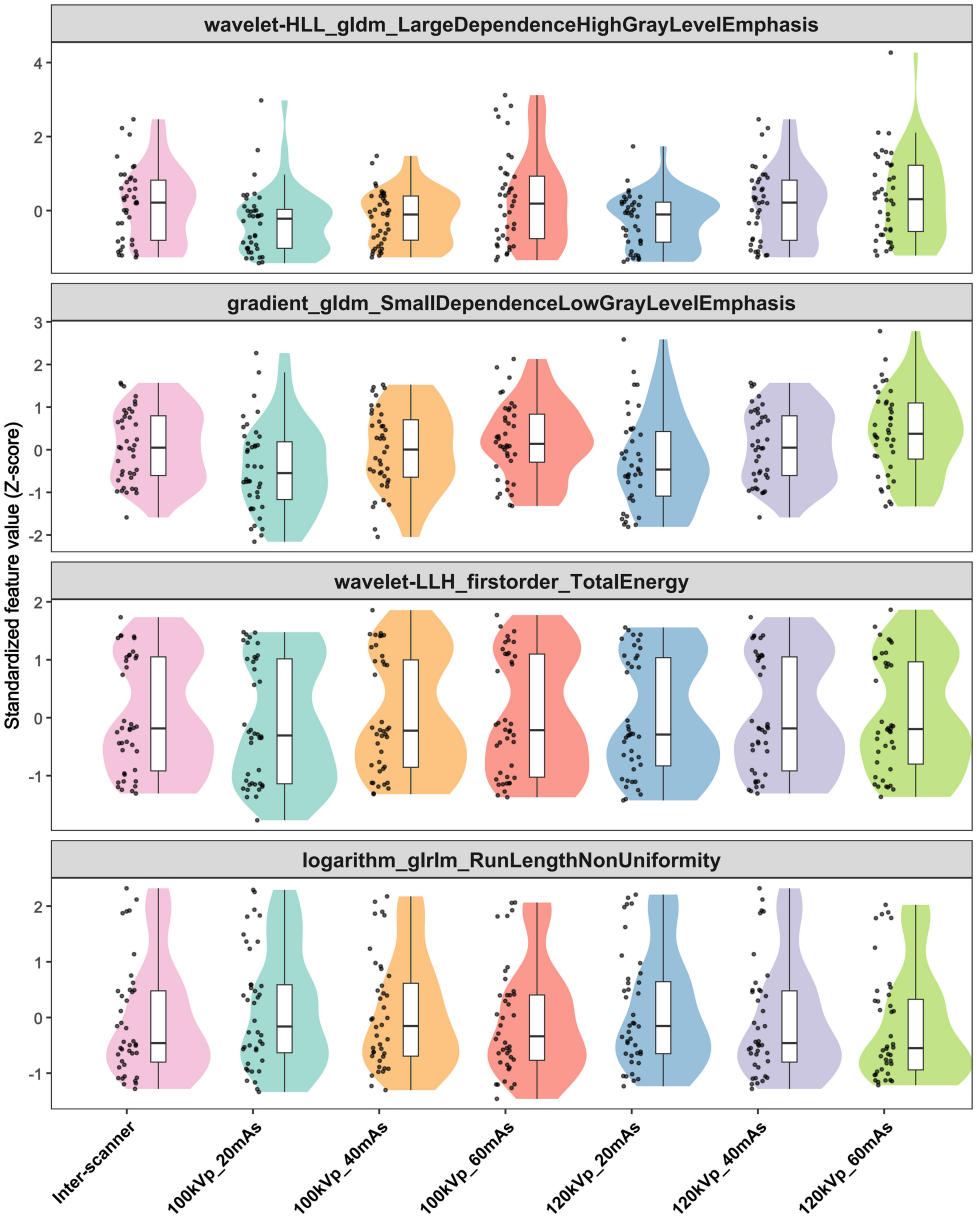
Stability patterns of randomly selected radiomic features across imaging conditions. The composite plot integrates violin plots, boxplots, and scatter points. The y-axis represents standardized feature values (Z-scores), and the x-axis represents imaging conditions. The top two rows display unstable features, whereas the bottom two rows display stable features.

To further illustrate the superior stability of representative radiomic features, two unstable and two stable features were randomly selected for visualization in **Figure 5**. The top two rows correspond to unstable features, whereas the bottom two rows represent stable features. As acquisition conditions varied along the x-axis, unstable features showed substantial fluctuations, reflected by changes in violin plot shapes and shifts in boxplot medians. In contrast, stable features showed minimal distributional variation, with largely consistent violin profiles and relatively stable median values across conditions.

### 3.4 Screening cohort validation

**Figure 6:**
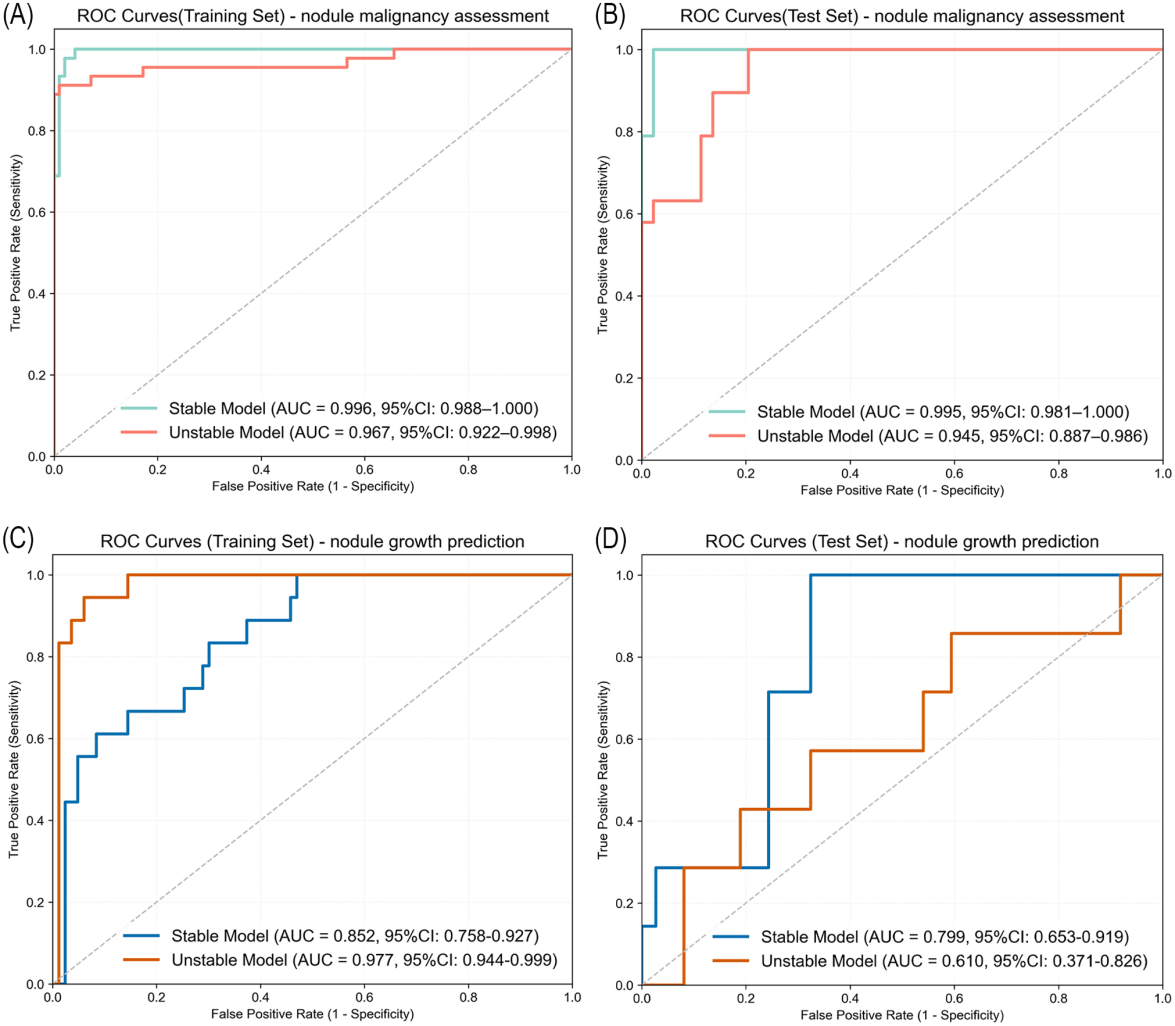
Performance of models built with stable and unstable radiomic features across two lung cancer screening datasets. **(A, B)** ROC curves of stable- and unstable-feature models for nodule malignancy assessment in the training and test sets, respectively. **(C, D)** ROC curves of stable- and unstable-feature models for nodule growth prediction in the training and test sets, respectively. ROC, receiver operating characteristic; AUC, area under the receiver operating characteristic curve; CI, confidence interval.

The performance of models constructed using stable and unstable features in the training and test sets across the two screening datasets is shown in **Figure 6**. For nodule malignancy assessment, the stable-feature model achieved a higher test AUC than the unstable-feature model (0.995 vs 0.945) and showed a smaller performance decline from training to test sets (*Δ*AUC = 0.001 vs 0.022). In the nodule growth prediction cohort, the stable-feature model yielded a higher test AUC than the unstable-feature model (0.799 vs 0.610). Interestingly, the stable-feature model exhibited lower performance variability across sets (*Δ*AUC = 0.053 vs 0.367), indicating improved model generalizability.

The predictive performance of individual representative stable features was further assessed in the two screening datasets. In the malignancy assessment dataset, all 13 representative stable features significantly differentiated malignant from benign nodules after false discovery rate correction (FDR-adjusted *p* < 0.05). Among these, six features achieved good discriminative performance, each with an AUC greater than 0.88. In the nodule growth prediction dataset, six of the 13 features showed significant differences between nodules with volume doubling time (VDT) < 400 and those with VDT > 600 following FDR adjustment (FDR-adjusted *p* < 0.05). The highest-performing feature in this cohort was log-sigma-2-0-mm-3D_glcm_Imc1, which achieved an AUC of 0.765 (95% CI, 0.671-0.850). Detailed results for each feature are provided in **Supplementary Table 2**.

## 4 Discussion

This study systematically evaluated the stability of radiomics features from lung nodules under LDCT lung cancer screening conditions, focusing on inter- and intra-scanner acquisition parameter variability. Our findings indicated that inter-scanner variability had a greater impact on feature stability than intra-scanner parameter adjustments; among intra-scanner variables, tube current changes generally affected stability more than tube voltage alterations. By intersecting inter-scanner and intra-scanner analyses and applying hierarchical clustering, 13 representative stable radiomics features were identified. These features exhibited consistent discriminative performance across two independent lung cancer screening datasets for malignancy assessment and nodule growth prediction. Models built using representative stable features achieved better performance on the test sets than models using unstable features, suggesting improved model generalization.

In the inter-scanner test, our results had a lower level of feature repeatability compared with those reported in previous studies (19, 21). Peng et al. reported a repeatability ratio of 20.5% (266/1295) in the inter-scanner test, whereas Jha et al. reported a repeatability ratio of approximately 30%. This discrepancy may be attributable to our use of a stricter ICC threshold for selecting stable radiomic features. By contrast, in the intra-scanner test, feature repeatability was higher than that reported in previous studies (19, 21). This may be explained by the fact that our analysis considered only tube voltage and tube current as the primary acquisition parameters, whereas prior studies included a broader range of parameters, such as slice thickness and reconstruction kernels.

The 292 stable radiomic features identified in both the inter-scanner and intra-scanner tests showed partial overlap with the stable features reported in previous phantom-based studies, suggesting a certain degree of consistency in radiomic feature stability across studies. Specifically, compared with the 19 representative stable features reported by Peng et al. (21), six overlapping features were observed, including original_firstorder_Energy, original_firstorder_Mean, square_firstorder_Median, squareroot_firstorder_Energy, logarithm_firstorder_Entropy, and lbp-2D_firstorder_Energy. These features are predominantly first-order statistics, and their stability may be related to their ability to capture global intensity characteristics of images. In addition, four of the eight stable features reported by Hertel et al. (17) were replicated in the present study: original_glszm_GrayLevelNonUniformityNormalized, original_firstorder_90Percentile, original_firstorder_RobustMeanAbsoluteDeviation and original_firstorder_Median. This alignment further supports the potential generalizability of such fundamental features across different phantoms and scanning conditions.

In lung cancer screening-based validation, the stable feature set identified in this study shared common features with those selected in previous radiomics studies. For example, in a volume doubling time-based analysis comparing nodules with values of 400 days or less and greater than 400 days, Ma et al. included original_glrlm_RunEntropy and original_shape_MeshVolume in their final predictive model, both of which were also identified as stable features in this study (37). Similarly, studies by Liu et al. (13) and Zyla et al. (14) focusing on nodule malignancy prediction incorporated original_firstorder_RootMeanSquared and original_firstorder_InterquartileRange into their respective feature sets, both of which were included among the stable features in this study. These overlaps suggest that phantom-validated stable features may exhibit not only reproducibility across acquisition conditions but also potential relevance for characterizing lung cancer screening datasets.

Several limitations of this study should be noted. First, although the phantom-based design allows effective control of confounding factors, it cannot fully replicate the complex physiological realities present in actual clinical settings. Specifically, real-world respiratory motion artifacts, cardiac pulsation, and varying patient body habitus, alongside underlying background pathologies such as emphysema, may introduce structural blurring and density alterations that further challenge radiomic feature stability. Second, the intra-scanner analysis focused primarily on tube voltage and tube current and did not account for other acquisition and reconstruction parameters. Reconstruction kernel, slice thickness, and iterative reconstruction algorithms are known to influence image spatial resolution and noise texture, and their effects on radiomic feature reproducibility were not captured in the present analysis. Future studies incorporating a broader range of reconstruction parameters would provide a more comprehensive characterization of feature stability under conditions representative of multicenter screening practice. Third, this study did not investigate the stability of radiomic features in part-solid nodules. Nevertheless, the phantom design incorporated eight simulated solid and ground-glass lung nodules with varying sizes and densities, reflecting common nodule types encountered in low-dose CT lung cancer screening. Fourth, this study assessed the predictive value of stable radiomic features only for common lung cancer screening tasks, including nodule malignancy assessment and growth prediction. The applicability of these features to other screening-related tasks, such as lesion aggressiveness risk prediction and individual-level risk stratification, warrants further investigation. In addition, the stability-informed validation framework in this study may offer a transferable methodological reference for CT-based radiomic characterization of incidental endocrine lesions co-detected during LDCT screening. For example, in the clinical management of adrenal incidentalomas, acquisition-stable feature reproducibility across different scanners is equally an important consideration for reliable non-invasive tumor stratification and clinical translation (23, 38, 39).

In conclusion, this study explored radiomic feature stability under lung cancer screening protocols and identified a subset of representative stable features through rigorous stability screening. These features showed consistent performance across different scanners and acquisition settings. When evaluated in independent lung cancer screening datasets, representative stable features achieved better predictive values than unstable features, and models constructed using representative stable features exhibited improved generalization performance. Overall, this study underscores the importance of considering feature stability in radiomics research and offers methodological insights that may support more reliable and standardized applications of radiomics in lung cancer screening.

## Data Availability

The original contributions presented in the study are included in the article/**Supplementary Material**. Further inquiries can be directed to the corresponding authors.
