# Supplementary material for: Phantom-based evaluation of radiomics feature stability for low-dose CT lung cancer screening
Source: Front Endocrinol (Lausanne). 2026 Jul 21;17:1880461. doi: 10.3389/fendo.2026.1880461 (PMC13433243; doi:10.3389/fendo.2026.1880461)
Supplement: Supplementary file 1 [file SupplementaryFile1.docx]

Supplementary Material

# 1 Supplementary Data

1. **Inclusion and exclusion criteria of lung cancer screening datasets**

**1.1 Cohort for Nodule Malignancy Assessment (n = 207)**

This cohort was derived from a lung cancer screening population and defined according to standardized imaging assessment and follow-up criteria.

Inclusion criteria were as follows:

(1) availability of baseline low-dose CT images with adequate image quality for radiomic analysis; and

(2) sufficient follow-up or pathological confirmation to allow reliable determination of nodule outcome.

Exclusion criteria included:

(1) incomplete follow-up;

(2) poor image quality precluding reliable feature extraction;

(3) prior antitumor therapy before outcome determination; and

(4) discordance between imaging findings and pathological results.

Based on outcome determination, nodules were subsequently classified as benign or malignant.

Nodules were classified as benign if they met one or more of the following criteria:

(1) stable or decreased size during longitudinal follow-up;

(2) imaging features consistent with benignity; and

(3) absence of imaging features suggestive of malignancy after completion of the recommended surveillance period, according to the Lung-RADS 2022 assessment system.

Nodules were classified as malignant if they met one or more of the following criteria:

(1) pathologically confirmed lung cancer;

(2) highly suspicious malignant imaging features identified on screening or diagnostic CT, with final confirmation by surgery or pathology.

All malignant nodules had not received preoperative antitumor therapy.

**1.2 Cohort for Nodule Growth Prediction (n = 145)**

This cohort was established to evaluate nodule growth dynamics using volume doubling time (VDT) and was derived from the same lung cancer screening population.

Inclusion criteria were as follows:

(1) availability of at least two chest CT examinations with a clearly defined interval and comparable imaging parameters;

(2) clear identification of the same pulmonary nodule on both scans with reliable volumetric segmentation; and

(3) a follow-up duration sufficient for VDT calculation.

Exclusion criteria included:

(1) poor image quality or failed nodule segmentation;

(2) antitumor therapy, infection, or other clinical interventions occurring between scans that could substantially affect nodule volume; and

(3) inability to confirm that nodules identified at different time points represented the same lesion.

# 2 Supplementary Figures and Tables

## 2.1 Supplementary Tables

**Supplementary Table 1**. Distributions of characteristics in the training and test sets across the two lung cancer screening datasets.

|  | **nodule malignancy assessment** | | **nodule growth prediction** | |
| --- | --- | --- | --- | --- |
|  | **Training** | **Test** | **Training** | **Test** |
| **Label** |  |  |  |  |
| **Label 1** | 45 (31.25) | 19 (30.16) | 18(17.82) | 7(15.91) |
| **Label 0** | 99 (68.75) | 44 (69.84) | 83(82.18) | 37(84.09) |
| **Gender** |  |  |  |  |
| **Male** | 81(56.25) | 35(55.56) | 58(57.43) | 25(56.82) |
| **Female** | 63(43.75) | 28(44.44) | 43(42.57) | 19(43.18) |
| **Age (years)** | 65.27±7.13* | 68.00±6.25* | 65.67±7.25* | 64.70±7.37* |

Label definitions: Label 1 denotes malignant nodules in the lung cancer screening dataset for malignancy assessment or nodules with a volume doubling time (VDT) < 400 in the lung cancer screening dataset for nodule growth prediction. Label 0 denotes benign nodules in the lung cancer screening dataset for malignancy assessment or nodules with VDT > 600 in the lung cancer screening dataset for nodule growth prediction. *Values refer to mean ± standard deviation.

**Supplementary Table 2.** Discriminative performance of each representative stable radiomic feature across two independent lung cancer screening datasets.

| **Representative stable radiomic features** | **FDR_P** | | **AUC (95%CI)** | |
| --- | --- | --- | --- | --- |
|  | **nodule malignancy assessment** | **nodule growth prediction** | **nodule malignancy assessment** | **nodule**  **growth prediction** |
| original_firstorder_TotalEnergy | < 0.001 | 0.029 | 0.894  (0.835-0.952) | 0.662  (0.520-0.793) |
| wavelet-LLH_firstorder_TotalEnergy | < 0.001 | 0.001 | 0.985  (0.973-0.995) | 0.730  (0.600-0.840) |
| logarithm_glrlm_RunLengthNonUniformity | < 0.001 | 0.002 | 0.976  (0.957-0.992) | 0.716  (0.588-0.830) |
| logarithm_glcm_Idmn | < 0.001 | 0.229 | 0.770  (0.688-0.841) | 0.594  (0.449-0.716) |
| logarithm_glrlm_GrayLevelNonUniformity | < 0.001 | 0.001 | 0.994  (0.986-0.999) | 0.729  (0.614-0.837) |
| logarithm_firstorder_Mean | 0.005 | 0.973 | 0.374  (0.287-0.477) | 0.502  (0.364-0.647) |
| log-sigma-4-0-mm-3D_glszm_GrayLevelNonUniformity | < 0.001 | 0.031 | 0.883  (0.825-0.940) | 0.656  (0.520-0.783) |
| log-sigma-4-0-mm-3D_ngtdm_Contrast | 0.025 | 0.452 | 0.401  (0.325-0.493) | 0.440  (0.306-0.566) |
| log-sigma-3-0-mm-3D_firstorder_10Percentile | < 0.001 | 0.452 | 0.250  (0.176-0.320) | 0.436  (0.309-0.562) |
| log-sigma-3-0-mm-3D_glcm_ClusterProminence | < 0.001 | 0.509 | 0.693  (0.623-0.767) | 0.550  (0.423-0.685) |
| log-sigma-5-0-mm-3D_ngtdm_Complexity | < 0.001 | 0.183 | 0.822  (0.758-0.880) | 0.605  (0.484-0.725) |
| log-sigma-2-0-mm-3D_glcm_ClusterShade | 0.025 | 0.572 | 0.598  (0.515-0.679) | 0.540  (0.422-0.653) |
| log-sigma-2-0-mm-3D_glcm_Imc1 | < 0.001 | < 0.001 | 0.994  (0.986-0.999) | 0.765  (0.671-0.850) |

AUC, area under the receiver operating characteristic curve; CI, confidence interval; FDR, false discovery rate.

## 2.2 Supplementary Figures

**Supplementary Figure 1.** (**A)** Feature-level ICC rank comparison in the Discovery CT750 HD (GE) scanner. (**B)** Feature-level ICC rank comparison in the SOMATOM Drive (Siemens) scanner. (**C)** Feature-level ICC rank comparison in the IQon Spectral CT (Philips) scanner. Each point represents a radiomic feature, with its position defined by the ICC-based robustness ranks for the two parameter variations. ICC, intraclass correlation coefficient.

**Supplementary Figure 2.** Additional inter-scanner distributional stability of radiomics features with high ICC values (ICC ≥ 0.95). Boxplots show the $-log₁₀(FDR-\mathrm{adjusted}p-values)$ from paired two-sided Wilcoxon signed-rank tests for the remaining five inter-scanner comparisons not presented in Figure 3. The horizontal dashed line indicates the FDR-corrected significance threshold ($\alpha$= 0.05).

**Supplementary Figure 3.** Hierarchical clustering dendrogram of 292 stable radiomic features based on Spearman correlation (distance threshold = 0.2).
